# 3HP preventive treatment among children and adolescents with HIV and child household contacts of TB patients

**DOI:** 10.5588/ijtldopen.24.0305

**Published:** 2024-09-01

**Authors:** A. Kinikar, R. Borse, B. Randive, P. Kamath, S.K. Mattoo, M. Parmar, H. Solanki, V. Mave, A. Gupta, R.E. Chaisson, N. Suryavanshi

**Affiliations:** ^1^ Byramjee Jeejeebhoy Government Medical College (BJGMC), Pune, India;; ^2^ BJGMC–Johns Hopkins University Clinical Research Site, Pune, India;; ^3^ Centre for Infectious Diseases in India, Johns Hopkins India, Pune, India;; ^4^ Central Tuberculosis Division, New Delhi, India;; ^5^ World Health Organisation, Country office for India, New Delhi, India;; ^6^ Johns Hopkins University, School of Medicine, Baltimore, MD, USA.

**Keywords:** tuberculosis, paediatric TB, TB preventive treatment, children and adolescents, HIV

## Abstract

**INTRODUCTION:**

India’s National TB Elimination Programme plans to roll out short-course TB preventive therapy (TPT) using 3 months of rifapentine and isoniazid (3HP). Understanding the feasibility and safety of children in programmatic settings is critical for widespread implementation. We present the findings of a targeted scale-up of 3HP among children and adolescents living with HIV (CALHIV) and child household contacts (>2 to <6 years) of pulmonary TB patients (CHHC).

**METHODS:**

Between December 2021 and July 2023, eligible CALHIV and CHHC participants were given weekly dosages of 3HP for 3 months at antiretroviral therapy (ART) and TB clinics, respectively, of a public hospital in Pune, India.

**RESULTS:**

Of 97 children screened, 91 initiated 3HP (32 CALHIV and 59 CHHC). The median age of CALHIV was 14 years; 66% were male and on dolutegravir-based ART. The median age of CHHC was 4 years; 47% were males. Thirty-one (97%) CALHIV and 56 (95%) CHHC completed 3HP without dolutegravir dose adjustment. None of the child participants discontinued 3HP due to adverse events. No child participant developed TB during 1 year of follow-up post-3HP.

**CONCLUSION:**

Our study provides evidence of the uptake and feasibility of the planned nationwide rollout of 3HP.

## RÉSUMÉ

**INTRODUCTION:**

Le programme national indien d'élimination de la tuberculose prévoit de mettre en place un traitement préventif de courte durée contre la tuberculose (TPT) en utilisant trois mois de rifapentine et d'isoniazide (3HP). Il est essentiel de comprendre la faisabilité et la sécurité des enfants dans le cadre des programmes pour pouvoir les mettre en œuvre à grande échelle. Nous présentons les résultats d'une extension ciblée de la 3HP parmi les enfants et les adolescents vivant avec le VIH (CALHIV, pour l’anglais « *children and adolescents living with HIV* ») et les enfants contacts familiaux (>2 à <6 ans) de patients atteints de tuberculose pulmonaire (CHHC, pour l’anglais « *child household contacts* »).

**MÉTHODES:**

Des doses hebdomadaires de 3HP ont été administrées pendant 3 mois aux participants éligibles CALHIV et CHHC dans les cliniques de thérapie antirétrovirale (ART) et de tuberculose, respectivement, d'un hôpital public de Pune, en Inde, entre décembre 2021 et juillet 2023.

**RÉSULTATS:**

Sur 97 enfants dépistés, 91 ont initié 3HP (32 CALHIV et 59 CHHC). L'âge médian des CALHIV était de 14 ans ; 66% étaient de sexe masculin et sous ART à base de dolutegravir. L'âge médian des CHHC était de 4 ans ; 47% étaient des hommes. Trente et un (97%) CALHIV et 56 (95%) CHHC ont terminé l'étude 3HP sans ajustement de la dose de dolutégravir. Aucun des enfants participants n'a interrompu le traitement 3HP en raison d'effets indésirables. Aucun enfant participant n'a développé de tuberculose au cours de l'année de suivi post-3HP.

**CONCLUSION:**

Notre étude fournit des preuves de l'adoption et de la faisabilité de la mise en œuvre prévue de 3HP à l'échelle nationale.

TB remains a global public health problem, with an enormous burden of disease estimated at 10.6 million new cases in 2022, with 6.3% having HIV co-infection and 12% being children between 0 and 14 years of age.^1^ Prevention of TB disease through the use of TB preventive therapy (TPT) is critical to reduce the burden of disease and mortality caused by TB and to achieve the End TB Strategy targets set for 2030 and 2035.^1^

TB disproportionally affects children and adolescents with HIV (CALHIV), and TB incidence is eight times higher in CALHIV compared to HIV-negative children.^2^ Although antiretroviral therapy (ART) reduces the incidence of TB in people living with HIV (PLHIV),^2^ CALHIV remain at high risk for TB even if they have normal CD4 counts and viral suppression.^2,3^ The elevated TB risk among CALHIV despite ART indicates the ongoing need for effective and widespread implementation of TPT, especially with shorter, safer, and effective regimens in this population.

Child household contacts (CHHC) of TB patients are also at a high risk of TB infection and disease due to prolonged and proximal exposure to adults with TB. In high TB burden settings, the prevalence of TB infection among CHHC is several-fold higher than in the general population.^4^ Furthermore, the incidence of TB disease is observed to be greatest in the first year after exposure.

Isoniazid preventive therapy (IPT) as TPT can reduce the risk of developing TB by 59% among children aged ≤15 years.^5^ A meta-analysis reported that if TPT is provided on time it could reduce the risk of developing TB disease by 63% among all CHHC and 85% among children with TB infection.^6^ However, the effectiveness of IPT is limited by the treatment completion rates of 30% to 64% due to the long duration of treatment.^7^ A systematic review of child contact management reported that IPT completion rates in 17 studies varied from 0% in South Africa to 94.5% in Gambia, with most studies reporting <50% treatment completion rates. Moreover, studies from India, the country with the highest global burden of TB, demonstrated that treatment completion rates among CHHC were 16% to 20%.^8,9^ Pill size, bitter taste, long duration of treatment, and perceived medication adverse effects were some of the identified treatment-related challenges.^10^

The WHO recently recommended TPT to household contacts of all ages.^11^ In 2018, the United Nations High-Level Meeting (UNHLM) set ambitious targets, committing to provide TPT to 4 million children <5 years old and 20 million household contacts ≥5 years old.^12^ In 2023, the UNHLM reported that only 1.6 million children and 0.6 million other household contacts were receiving TPT, and therefore much more needs to be done.^13^

One of the TPT regimens recommended by the WHO is the shorter 3 months of once-weekly rifapentine (P, RPT) and isoniazid (H, INH), known as 3HP. Evidence shows that the 3HP regimen is effective and is well tolerated with better adherence rates.^14,15^ In line with the WHO recommendation, India updated its TPT policy.^16^ India’s National Tuberculosis Elimination Programme (NTEP) currently recommends that CALHIV and CHHC be actively screened for TB and be offered TPT, including 3HP (by age and weight band) to CHHC ≥2 years of age and ≥10 kg weight and plans are now underway to scale up 3HP nationally. Moreover, on World Tuberculosis Day, the Prime Minister addressed the One World TB Summit held in Varanasi, India, and announced the official pan-India rollout of shorter TPT regimens.^17^

Although 3HP is reported to be safe and effective under clinical trial conditions, understanding the uptake and feasibility of implementation under programmatic conditions is needed. Hence, this study demonstrates the targeted scale-up of 3HP for CALHIV and CHHC aged ≥2 years to <6 years in India.

## METHODS

This study was conducted under the IMPAACT4TB consortium as a demonstration project between December 2021 and July 2023 at an ART and an NTEP centre of a tertiary care public hospital, Byramjee Jeejeebhoy Government Medical College (BJGMC) and Sassoon General Hospital, Pune, India. The study aimed to provide important data on the feasibility and uptake of 3HP to scale up under HIV and TB programmatic settings. The study protocol was approved by the BJGMC Ethics Committee, Pune, India; and the Johns Hopkins Medicine Institutional Review Board, Baltimore, MD, USA.

We screened CALHIV accessing care at the ART centre and CHHC between 2 and 6 years of index sputum acid-fast bacilli (AFB) positive pulmonary TB patients who had been on TB treatment for up to 2 months duration and were seeking care at the TB centre. Children were screened for TB and underwent baseline laboratory assessments. Those who had normal blood parameters (serum bilirubin, aspartate aminotransferase, alanine aminotransferase, complete blood count, blood urea, serum creatinine) were enrolled in the study after written informed consent from caregivers and assent from child participants was obtained. Oral assent was obtained from participants aged 7 to <12 years. Written assent was obtained from participants aged 12 to <18 years. Counsellors counselled and educated caregivers and participants on 3HP regimen-specific information, adherence, medication adverse effects and TB symptoms.

### 3HP administration

Upon enrolment, participants received their first dose of 3HP by self-administration or administration by a caregiver with observation by study nurses. Caregivers were given the remaining month’s supply (three weekly doses) to be administered by the caregiver at home with weekly telephone reminders by the staff compared to monthly telephone calls in routine care. Doses were dispensed monthly for the remaining 2 months, combining 3HP study visits with ART visits where applicable. The study counsellor conducted phone check‐ins with caregivers weekly for 3 months to monitor for 3HP adherence, any adverse event (AE) defined as any untoward medical occurrence in a patient that does not necessarily have a causal relationship with the treatment and TB symptoms. We used The Division of AIDS (DAIDS) Table for Grading the Severity of Adult and Pediatric Adverse Events, Version 2.1, as a reference for grading AE severity.^18^ Caregivers were instructed to report any illness or symptoms immediately after consuming the 3HP dose to the study clinician via the contact number provided on the participant’s study identification card. TB incidence and AEs were also monitored during the 3HP treatment at monthly visits at the ART centre and TB clinic, and for TB symptoms at Months 6 and 12, in-person visits post-enrolment. The fixed-dose combinations (FDCs) of isoniazid (H) and rifapentine (P) were used for CALHIV participants > 14 years of age and weight >30 kg, and pyridoxine (50 mg) weekly was given along with the 3HP dosage. CALHIV <30 kg of weight and CHHC were given weight-band-wise dosage as singles of P-150 mg and H-100 mg. The number of tablets consumed was 5 to 12 based on the child’s weight. The parent/caregiver of the child was informed about the weekly drugs that are expected to be consumed by them and the return of the empty blister packs on the next monthly visit. Any challenges faced by caregivers/participants while 3HP administration at home were noted by counsellors in the progress notes.

### Statistical analysis

We performed descriptive analysis to present the demographic characteristics of the study populations, as well as the safety, tolerability, and effectiveness of 3HP. The proportion of participants who discontinued 3HP due to drug toxicity among those who received at least one dose of 3HP was assessed for 3HP safety or tolerability. The 3HP treatment completion was assessed as the proportion of participants who completed twelve dosages of 3HP in sixteen weeks. The incidence of TB among those who completed at least 11 doses of 3HP was used to assess the effectiveness of 3HP over the 12 months of follow-up. Analysis in this manuscript is restricted to CALHIV up to 18 years of age and CHHC of index sputum AFB-positive TB patients <6 years of age.

## RESULTS

As shown in the Figure, we approached 201 potential participants in both groups: 60 CALHIVs taking treatment from the ART centre at BJGMC and 141 CHHC from the TB clinic of BJGMC and other TB clinics around Pune City to assess their willingness and eligibility to participate in the study. Of these, 97 were eligible/willing to participate and were screened; 91 (94%) were enrolled and started on 3HP (32 CALHIV and 59 CHHC). Among 32 CALHIV, 21 (65.6%) were males, with a median age of 14 years (interquartile range [IQR] 10.5–15.5), median time on ART 2,185 days (IQR 1,375–2,530), and median CD4 count of 737 (IQR 511–866) and viral load (VL) was undetectable among 23 (76.7%). Among 59 CHHC, the median age was 4 years (IQR 3–5), and 28 (47.5%) were males (Table 1).

**Figure.**
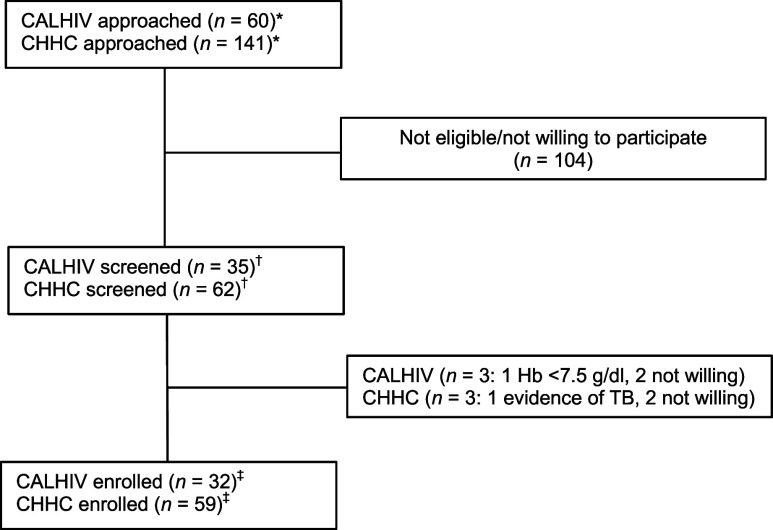
CONSORT diagram of screening and enrolment of study participants in 3HP preventive treatment study. *Caregivers of CALHIV started on ART at BJGMC, and CHHCs of index TB patients were approached. ^†^Caregivers of CALHIV and CHHC who provided consent for participation were screened and evaluated for eligibility were included as screened. ^‡^Those who were eligible and willing were enrolled. CALHIV = children and adolescents living with HIV; CHHC = child household contacts; 3HP = weekly isoniazid-rifapentine for 3 months; CONSORT = Consolidated Standards of Reporting Trials.

**Table 1.** Baseline demographic and clinical characteristics of CALHIV and CHHC enrolled in 3HP preventive treatment demonstration study in Pune, India.

	CALHIV	CHHC
	*n* (%)	*n* (%)
Age, years, median [IQR]	14 [10.5–15.5]	4 [3–5]
Male sex	21 (65.6)	28 (47.5)
CD4 count, cells/mm^3^, median [IQR]	737 [511–866]	NA
HIV viral load not detectable	23 (76.7)	NA
Dolutegravir-based ART (TLD, ZLD)	21 (65.6)	NA

CALHIV = children and adolescents living with HIV; CHHC = child household contacts; 3HP = weekly isoniazid-rifapentine for 3 months; IQR = interquartile range; NA = not available; ART = antiretroviral therapy; TLD = tenofovir disoproxil, lamivudine, dolutegravir; ZLD = zidovudine + lamivudine + dolutegravir.

### 3HP treatment completion and adverse events

Overall, 91 child participants were initiated on 3HP, and 87 (95.6%) completed at least 11 of 12 doses within 16 weeks. Treatment completion was 97% among CALHIVs and 95% among CHHCs. No serious AEs requiring hospitalisation or treatment interruption due to AEs were observed among these 91 children. None of the child participants developed TB during the 12-month follow-up period. All AEs were classified as Grade 1, and no serious adverse events occurred during treatment, either among CALHIVs or CHHCs. All the symptoms resolved during the study follow-up.

### 3HP treatment completion and adverse events among CALHIVs

Among 32 CALHIVs enrolled, 7 (22.5%) reported mild AEs (Table 2). The most common AE was gastrointestinal (GI) symptoms reported by 3 (9%) children. One child reported two episodes of GI symptoms, and two experienced one episode of GI symptoms associated with either the first or the second dose. GI symptoms among CALHIV were nausea and vomiting, which were resolved within one day. Two (6%) CALHIV experienced flu-like symptoms, consisting of fever, dizziness and vomiting for one day; one participant had two episodes associated with the first two doses of 3HP in Month 1, while the other occurred with the 10^th^ dose in Month 3. One child (3%) reported hypersensitivity and 1 (3%) peripheral neuropathy. None of the seven children discontinued study medication due to these symptoms. All these grade-one events were managed by the study clinician on time. Of 32 CALHIV, one participant could not complete 3HP treatment due to loss to follow-up (changed residence).

**Table 2.** Treatment completion and adverse events among participants initiated on 3HP preventive treatment.

Characteristic	CALHIV	CHHC
	*n* (%)	*n* (%)
3HP initiated	32	59
3HP completed^*^	31 (97)	56 (95)
Adverse events	7 (22.5)	5 (8)
Flu-like symptoms	2 (6)	5 (8)
Gastro-intestinal	3 (9)	3 (5)
Hepatotoxicity	0	0
Hypersensitivity	1 (3)	1 (1.7)
Peripheral neuropathy	1 (3)	0
Other	2 (6)	1 (1.7)
Serious adverse events requiring hospitalisation	0	0
Drug discontinuation due to adverse events	0	0
3HP not completed due to adverse event mild fever (parents decided to discontinue)	0	1
3HP not completed due to loss to follow-up	1	2

* Includes those who have completed at least 11 doses of 3HP within 16 weeks of the first dose.

3HP = weekly isoniazid-rifapentine for 3 months; CALHIV = children and adolescents living with HIV; CHHC = child household contacts.

### 3HP treatment completion and adverse events among CHHCs

Among CHHCs, 5 (8%) reported flu-like symptoms of fever and dizziness. Of these five, one had only flu-like symptoms resolving within one day; 3 also reported GI symptoms, of which two had two episodes, and one had three episodes with their first two doses, all resolving within 2 days; and one reported three hypersensitivity reactions in the form of skin rash after the first three doses, resolving within a day.

Of 59 CHHCs, three participants discontinued 3HP treatment due to non-adherence to the treatment schedule. One of the participants developed a mild fever; hence, the parents did not continue the treatment. Two caregivers refused to continue treatment further due to the inability to come for scheduled follow-ups. Although adherence to 3HP was good, caregivers of CHHCs reported challenges with the number of pills to be consumed by young children.

## DISCUSSION

Scaling up shorter TPT regimens such as 3HP is one of the key priorities for global TB elimination and the NTEP to meet the sustainable development goals of End TB targets. This study demonstrated that 3HP can be safely implemented with high adherence among CALHIV and CHHCs, two of the most vulnerable populations. To the best of our knowledge, this is the first study to provide much-awaited evidence on the uptake, tolerability, and completion of 3HP among CALHIV and CHHCs in the context of routine HIV and TB care, thus providing crucial evidence to inform the nationwide scale-up of 3HP TPT among CALHIV and CHHCs.

Scaling up any drug intervention in the public health domain requires a demonstration of comparable outcomes of clinical trials and programmatic implementation in routine programmatic settings. A small 3HP tolerability study among CHHCs in China reported that 10 of 26 children (38.5%) experienced Grade 1 AEs.^19^ This is almost four times higher than observed in our study (8%). However, no child required discontinuation of 3HP, as also observed in other studies.^19–21^

It is important to note that despite few Grade 1 AEs, these participants completed the study treatment of 3HP. Our CALHIVs were on a dolutegravir-based ART regimen when 3HP TPT was concurrently administered. Although our study was not a pharmacokinetic study to show the interaction between 3HP and dolutegravir, it is important to note that a recent pharmacokinetics study of 3HP among PLHIV recommends that 3HP can be given to PLHIV taking dolutegravir-based ART without dose adjustments.^22^ However, in children, the interaction between 3HP and dolutegravir-based ART is currently being studied by the DOLPHIN kid’s study. CALHIV in our study were on ART for a long time with a median days on ART of 2,185 days, which is comparable to a randomised trial done among PLHIV in Uganda.^23^

Our study showed a low rate of minor AEs in children who started 3HP in a real-life context. Additionally, the study provided evidence for safety and a high rate of adherence to the treatment. However, it remains crucial to monitor children on 3HP to observe unexpected AEs periodically.

Participants in our study demonstrated high adherence to 3HP. Counselling and weekly phone calls compared to monthly phone calls in routine care motivated parents/caregivers to continue and complete the study medication for the prescribed period. Some caregivers reported difficulty for children to take 5–12 tablets of each dose; children under 5 years have difficulty swallowing capsules. High pill burden warrants the need for child-friendly formulations such as dispersible FDCs that would reduce pill burden, maintain good adherence, and improve the patient’s overall treatment experience.

Our study has some limitations. As the sample size of our study is small, it is possible that it may not have captured the occurrence of rare drug-related adverse effects. Second, being an implementation project, we did not compare the performance of the 3HP regimen with other TPT regimens. However, available evidence suggests that it is an effective regimen for the prevention of TB in children.^7,15^ We did not capture any cost-related data; however, other cost-effective modelling analyses among children reported household contact investigation with the provision of short-course TPT is highly cost-effective.^24–27^

## CONCLUSION

Our study provides much-needed evidence for the planned nationwide rollout of 3HP in India with high uptake, tolerability, feasibility, and adherence to 3HP among children.

## References

[1] World Health Organization. Global tuberculosis report, 2023. Geneva, Switzerland: WHO, 2023.

[2] Dodd PJ, The impact of HIV and antiretroviral therapy on TB risk in children: a systematic review and meta-analysis. Thorax. 2017;72(6):559–575. 28115682 10.1136/thoraxjnl-2016-209421 PMC5520282

[3] Frigati LJ, Tuberculosis infection and disease in South African adolescents with perinatally acquired HIV on antiretroviral therapy: a cohort study. J Int AIDS Soc. 2021;24(3): e25671. 33719199 10.1002/jia2.25671 PMC7957181

[4] Tiruneh F, Deyas Y. How far does highly active antiretroviral treatment reduce TB incidence among children? A marginal structural modeling analysis, Southwest Ethiopia. Ethiop J Health Sci. 2020;30(5):653–660. 33911825 10.4314/ejhs.v30i5.3 PMC8047272

[5] Ayieko J, Efficacy of isoniazid prophylactic therapy in prevention of tuberculosis in children: a meta-analysis. BMC Infect Dis. 2014;14(1):91. 24555539 10.1186/1471-2334-14-91 PMC3936889

[6] Martinez L, The risk of tuberculosis in children after close exposure: an individual-participant meta-analysis including 137,647 children from 46 cohort studies. Lancet Lond Engl. 2020;395(10228):973–984. 10.1016/S0140-6736(20)30166-5 PMC7289654 32199484

[7] Sterling TR, Three months of rifapentine and isoniazid for latent tuberculosis infection. N Engl J Med. 2011;365(23):2155–2166. 22150035 10.1056/NEJMoa1104875

[8] Shivaramakrishna HR, Isoniazid preventive treatment in children in two districts of South India: does practice follow policy? Int J Tuberc Lung Dis. 2014;18(8):919–924. 25199005 10.5588/ijtld.14.0072 PMC4589200

[9] Singh AR, Isoniazid preventive therapy among children living with tuberculosis patients: is it working? A mixed-method study from Bhopal, India. J Trop Pediatr. 2017;63(4):274–285. 28082666 10.1093/tropej/fmw086 PMC5914486

[10] Szkwarko D, Child contact management in high tuberculosis burden countries: a mixed-methods systematic review. PloS One. 2017;12(8):e0182185. 28763500 10.1371/journal.pone.0182185 PMC5538653

[11] World Health Organization. Latent tuberculosis infection: updated and consolidated guidelines for programmatic management. Geneva, Switzerland: WHO, 2023. 30277688

[12] United Nations. Fight to end tuberculosis | General Assembly of the United Nations. New York, NY, USA: UN, 2018.

[13] World Health Organization. UN General Assembly High-level Meeting on the fight against tuberculosis, 2023. Geneva, Switzerland: WHO, 2023.

[14] Villarino ME, Treatment for preventing tuberculosis in children and adolescents. JAMA Pediatr. 2015;169(3):247–255. 25580725 10.1001/jamapediatrics.2014.3158 PMC6624831

[15] Sterling TR, Three months of weekly rifapentine and isoniazid for treatment of *Mycobacterium tuberculosis* infection in HIV-coinfected persons. AIDS Lond Engl. 2016;30(10):1607–1615. 10.1097/QAD.0000000000001098 PMC4899978 27243774

[16] Central TB Division. Guidelines for programmatic management of tuberculosis preventive treatment in India. New Delhi, India: Ministry of Health and Family Welfare, 2023.

[17] Press Information Bureau. PM addresses One World TB Summit in Varanasi, Uttar Pradesh. New Delhi, India: Government of India, 2023.

[18] U.S. Department of Health and Human Services, National Institutes of Health, National Institute of Allergy and Infectious Diseases, Division of AIDS. Division of AIDS (DAIDS) Table for Grading the Severity of Adult and Pediatric Adverse Events, Corrected Version 2.1. Bethesda, MD, USA: NIH, 2017.

[19] Yang H, High rate of completion for weekly rifapentine plus isoniazid treatment in Chinese children with latent tuberculosis infection: a single center study. PloS One. 2021;16(6):e0253159 34115804 10.1371/journal.pone.0253159 PMC8195436

[20] Belknap R, Self-administered versus directly observed once-weekly isoniazid and rifapentine treatment of latent tuberculosis infection: a randomized trial. Ann Intern Med. 2017;167(10):689–697. 29114781 10.7326/M17-1150 PMC5766341

[21] Walker RE, Evaluation of 3 months of once-weekly rifapentine and isoniazid for latent tuberculosis infection. Ann Pharmacother. 2020;54(5):457–463. 31729245 10.1177/1060028019888855

[22] Dooley KE, Once-weekly rifapentine and isoniazid for tuberculosis prevention in patients with HIV taking dolutegravir-based antiretroviral therapy: a phase 1/2 trial. Lancet HIV. 2020;7(6):e409. 10.1016/S2352-3018(20)30032-1 32240629

[23] Semitala FC, Comparison of 3 optimized delivery strategies for completion of isoniazid-rifapentine (3HP) for tuberculosis prevention among people living with HIV in Uganda: a single-center randomized trial. PLOS Med. 2024;21(2): e1004356. 38377166 10.1371/journal.pmed.1004356 PMC10914279

[24] Jo Y, Cost-effectiveness of scaling up short-course preventive therapy for tuberculosis among children across 12 countries. eClinical Medicine 2021;31:100707. 10.1016/j.eclinm.2020.100707 PMC7846666 33554088

[25] Lung T, Household contact investigation for the detection of tuberculosis in Vietnam: economic evaluation of a cluster-randomised trial. Lancet Glob Health. 2019;7(3):e384. 10.1016/S2214-109X(18)30520-5 30784638

[26] Sekandi JN, Cost-effectiveness analysis of community active case finding and household contact investigation for tuberculosis case detection in urban Africa. PLoS One. 2015;10(2):e0117009. 25658592 10.1371/journal.pone.0117009 PMC4319733

[27] Mandalakas AM, Modelling the cost-effectiveness of strategies to prevent tuberculosis in child contacts in a high-burden setting. Thorax. 2013;68(3):247–255. 22717944 10.1136/thoraxjnl-2011-200933 PMC11967563

